# Toward Robust Electrical Heating Textiles: Factors Influencing Electrical Heating Performance and Durability

**DOI:** 10.1021/acsomega.5c06733

**Published:** 2025-10-20

**Authors:** Babak Abdi, Ali R. Tehrani-Bagha

**Affiliations:** School of Chemical Engineering, 174277 Aalto University, 02150 Espoo, Finland

## Abstract

The increasing demand for high-performance and durable e-textiles has driven the exploration of conductive coatings that combine electrical functionality with mechanical robustness. This work presents the development of conductive inks for e-textile applications, focusing on how binder selection, coating architecture, and MWCNT loading govern critical performance factors, including wettability, conductivity, Joule heating, mechanical durability, and bending resistance. The sample surfaces were examined using field emission scanning electron microscopy before and after washing and rubbing cycles. The biobinder created a hydrophilic surface with improved conductivity and enhanced electrical heating performance. The coated fabric showed increased resistance to bending, reduced bending durability, and lower washing and rubbing fastness. Meanwhile, the A-5001 binder exhibited higher resistance, lower conductivity, and lower temperatures under voltage but provided better washing and rubbing fastness, bending durability, and improved flexibility. We examined a hybrid approach using both binders, with the A-5001 binder applied in the first and third layers to improve durability and flexibility and the OC-Biobinder used as the middle layer to enhance conductivity and Joule heating. This approach utilizes the complementary properties of both the binders, enhancing the performance and providing a robust pathway toward durable and functional e-textiles.

## Graphical abstract

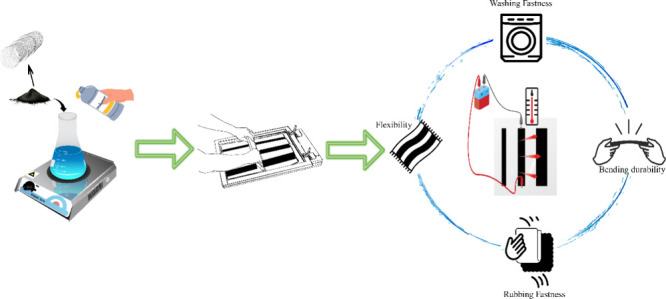

## 1 Introduction

The need for miniaturization, enhanced performance, and flexible wearable electronics underscores the demand for novel manufacturing processes and materials.^1,2^ Textiles are attractive substrates for printed electronics due to their flexibility, body compatibility, cost-effectiveness, and nontoxicity.^3–6^ Their potential for recyclability and, in the case of natural or biobased fibers, renewable and biodegradable properties have garnered significant attention in the field of wearable electronic technology, leading to the development of smart textiles.^7^ Electronic textiles (E-textiles) are increasingly valued for their unique electrical, thermal, and optical properties,^8–12^ with applications spanning sensing stimuli, energy harvesting and storage, personal thermal management, and aerospace technologies.^6,13–20^

Central to these advancements are conductive inks, which enable the integration of additional functionalities into textiles by providing electrical conductivity and low-resistance pathways.^7,21^ Typically, these inks are created by incorporating metal particles to achieve the necessary conductive properties. Despite their significance, conductive materials like gold, silver, and copper are often criticized for their substantial energy consumption, carbon emission, overuse of natural resources, and environmental pollution.^12^ However, carbon-based nonmetallic conductive particles, such as multiwalled carbon nanotubes (MWCNTs) and graphene nanoplatelets, have recently been prioritized over metals such as silver^22^ and copper^23^ due to their lower cost and functional advantages.^24–27^

Knife-edge coating (also known as knife-overroll coating) is a widely used technique for applying liquid formulations onto flexible substrates such as textiles. In this process, a liquid ink or dispersion is deposited by a sharp blade positioned at a controlled distance from the moving substrate, allowing for precise adjustment of coating thickness. The method offers several advantages, including simplicity of operation, compatibility with a wide range of viscosities and filler-loaded inks, uniform film formation even on porous surfaces, and good reproducibility. Importantly, knife-edge coating is highly scalable as the same principle is already established in large-scale roll-to-roll finishing lines used in paper, packaging, and textile industries, enabling efficient multilayer coatings with controlled thickness and minimal material waste.^28^

A clear and pressing need exists for the utilization of environmentally friendly materials, where possible, and discovery of innovative materials, and the industry is increasingly focused on developing sustainable ink chemistries. This includes reducing the use of solvents that release volatile compounds, adopting water-based ink media, which are perceived as more sustainable and cost-effective, and exploring energy-curable products.^12,29^ Furthermore, sustainable production demands the optimal utilization of energy, water, and other natural resources in the manufacturing process.^30^ The importance of using less environmentally harmful materials has been highlighted. However, these “eco-friendly alternatives” often underperform compared to traditional fossil-fuel-based or nonbiodegradable materials. Consequently, ongoing research^31–33^ is focused on understanding material perception and interchangeability to enhance user experiences. This allows manufacturers to confidently adopt eco-friendly alternatives to fossil-fuel-based plastics.^12^

Smart textiles are anticipated to experience remarkable advancements in the future. This renewed interest has led to the expansion of industrial goods in the sector.^34^ Despite more than 2 decades of development, smart textiles have yet to achieve widespread market adoption. Enhancing their poor usability is essential for improving user acceptance.^35,36^ The primary obstacle to the mass production of electrically conductive textiles today is ensuring the wash and wear durability of conductive compounds while maintaining their electrical performance.^15,37^ The growing demand for sustainability necessitates that the integrated conductive and electronic components possess washability comparable to that of their textile substrates. From a sustainability viewpoint, it is essential to ensure that the system remains functional and in use for as long as possible, thereby reducing the need for replacement.^38^

Exploring innovative active electrical heating textiles is crucial due to the growing demand for stretchable, wearable, and versatile thermal management solutions. Potential applications include personal thermal management in smart jackets and blankets, medical wearables for therapeutic heat, and improved thermal control in aerospace and automotive systems.^39^ Therefore, the significant potential demand for heating textiles and thermal garments in daily life has driven various research and development efforts.^40^ Electrical heaters convert electrical energy into thermal energy to maintain a suitable temperature for the human body. As an advanced personal heat management solution, electrical heating garments can regulate heat transfer between the human body and the external environment through conduction and radiation or provide active heating through Joule heating. Joule heating, also known as ohmic heating, is the process of generating heat by passing an electrical current through a medium (either liquid or solid) that has limited conductivity.^41^ Joule heating elements function as the operational components in electrical heaters.^42^ As current flows through conductive materials, heat is generated under an applied voltage due to inelastic collisions between accelerated electrons and phonons.^43^ Electrical heating textiles cater to personalized needs in diverse environments such as outdoor adventures, winter sports, and indoor offices.^39^ In addition, heating fabrics are increasingly being applied in biomedical and cosmetic fields, offering benefits for wound healing.^4,44^

This study addresses a significant research gap by focusing on the production of conductive aqueous inks for coated textiles. It provides a detailed comparison of a biobinder versus a commercially available PU binder, examining their impact on surface properties, electrical conductivity, Joule heating, washing and rubbing fastness, bending durability, and flexibility. This study offers insights into how different binder systems can be leveraged to create highly conductive and durable printed textiles.

## 2 Experimental Section

### 2.1 Materials

A knitted fabric, composed of 70% rayon and 30% organic cotton, weighing 175 g/m^2^ and yarn count of 60 Tex was sourced from Coveross company in Finland. MWCNTs with diameters of 50–85 nm, lengths of 10–15 μm, and a carbon content exceeding 94%, were purchased from Graphene Laboratories Inc. OC-Biobinder Lily 1450 (OC), an opaque water-based liquid with a solid content of ca. 27% and structural composition of L-(+)-lactic acid, (2S)-2-hydroxypropanoic acid with 1,2 benzisothiazol, and 5-chloro-2-methyl-3­(2H)-isothiazolone with 2-methyl-3­(2H)-isothiazolone as preservatives,^45^ was purchased from OrganoClick AB Company in Sweden. A polyurethane (PU)-based binder (A-5001, aroma-free, and hydrocarbon-rich weighted paste) suitable for printing textiles was obtained from Wennström company in Finland.

### 2.2 Fabrication Process

#### 2.2.1 Coating Ink Formulation

After conducting several preliminary screening tests, six distinct coating ink formulations were developed, each varying in binder type and functional particle concentration. To achieve this, three different concentrations of MWCNT were dispersed in 10 mL of water by stirring for 10 min followed by 25 min sonication at room temperature. Subsequently, the selected binder was added slowly to the solution and stirred for 1 h. This process resulted in six different coating inks with MWCNT concentrations of 9.1, 16.7, and 23.1 wt %, each using one of the two binders.

#### 2.2.2 Fabrication Process

The prepared inks were used to coat three different rectangular-shaped patterns on the fabric using the knife-edge coating method. For this, an open frame was used, three rectangular patterns were designed, and the rest of the surface was covered to prevent penetration of the ink from other parts of the frame. These three patterns were in the size of 0.5 × 11 cm^2^, 1 × 11 cm^2^, and 2 × 11 cm^2^. The ink was applied to the fabric and spread evenly with a squeegee through the open frame. This method ensured a uniform and controlled coating layer across the fabric surface. The printed samples were dried after each coating process according to the recommended conditions of the suppliers. Specifically, the coated samples with the OC bio binder and the A-5001 binder were dried after each coating process at 120 and 150 °C for 3 min. After the final coating layer was applied, the samples were dried at the same temperatures for 10 min. Subsequently, the coated samples were hot-pressed at 165 °C under a pressure of 150 kPa for 10 min. Figure S1 (See the Supporting Information section) shows the final printed patterns.

Table 1 shows the sample codes and coating formulation of the prepared samples. To study the effect of MWCNT concentration, the number of coating layers was kept the same on four layers, and three concentrations of 9.1, 16.7, and 23.1% were produced. Samples O-9.1%-4t, O-16.7%-4t, and O-23.1%-4t containing the biobinder and A-9.1%-4t, A-16.7%-4t, and A-23.1%-4t containing A-5001 were characterized in this study. The hybrid sample was prepared using a sequential coating approach with two different inks, each containing a 16.7 wt % MWCNT concentration. For this sample, the coating ink with the A-5001 binder was applied for the first and third layers, while the middle layer was coated with the ink containing the OC-biobinder.

**Table 1:** Sample Code and Coating Formulation

sample code	type of binder	number of the coating layers	MWCNT concentration (wt %)
O-9.1%-4t	OC-biobinder	4	9.1
O-16.7%-2t	OC-biobinder	2	16.7
O-16.7%-3t	OC-biobinder	3	16.7
O-16.7%-4t	OC-biobinder	4	16.7
O-23.1%-4t	OC-biobinder	4	23.1
A-9.1%-4t	A-5001	4	9.1
A-16.7%-2t	A-5001	2	16.7
A-16.7%-3t	A-5001	3	16.7
A-16.7%-4t	A-5001	4	16.7
A-23.1%-4t	A-5001	4	23.1
hybrid	A-5001 + OC-biobinder	3	16.7

### 2.3 Characterization

All specimens were conditioned before characterization in a standard textile testing atmosphere (20 ± 2 °C and 65 ± 4% RH) for a minimum of 24 h. This conditioning was applied prior to measurements of thickness, bending properties, electrical conductivity, and rubbing fastness.

#### 2.3.1 Thickness

A Lorentzen & Wettre SE 250 D (Finland) thickness tester was used to assess the thickness of the textile samples. This instrument features a micrometer resolution and an indication error of ±1 μm or 0.1% reading. The textile samples’ thickness was measured with a circular pressure foot, a test area of 10 cm^2^, and a test pressure of 20 kPa. Ten measurements were conducted on different parts of each sample, and the mean and standard deviation are reported.

#### 2.3.2 Bending Test

The bending resistance and bending stiffness of the samples were measured using the Lorentzen & Wettre SE 160 (Finland) bending tester. The bending resistance and bending stiffness were measured for a bending length of 25 mm. Each sample was clamped with the grip and deflected to the specified angle. The bending resistance was measured in all angle ranges of 0–5°, 0–7.5°, 0–15°, and 0–30°, and the bending stiffness was measured in an angle range of 0–7.5°. The samples were analyzed 10 times, and the average value with the standard deviation was reported.

#### 2.3.3 Scanning Electron Microscopy

The surface morphology and microstructure of the coated textile samples were examined using scanning electron microscopy (SEM). The textile samples were cut into small sections and mounted on aluminum stubs using double-sided conductive carbon tape to ensure proper adhesion and conductivity. To prevent charging and improve image quality, all samples were coated with a thin layer of gold/palladium (80/20) before scanning. SEM imaging was performed using emission SEM (Zeiss Sigma VP, Germany) equipped with an InBeam detector. The scanning was regulated in the high vacuum mode at an accelerating voltage of 20 kV. The samples were systematically scanned, and images were captured at various magnifications to examine the surface morphology, coating uniformity, and particle distribution in different stages after printing, after 5 washing cycles, and after 10 rubbing cycles and to understand the impact of different ink formulations on the final properties of the samples.

The quantitative analysis was done on the surface morphology of the SEM images of the coated sample with ImageJ. In this respect, the high-resolution images were selected and processed using ImageJ. The images were converted to 8-bit grayscale and thresholded to distinguish conductive particle agglomerates from the background. Then, the “Analyze Particles” function was used to determine parameters such as the particle count, the total and average area, and the percentage area coverage.

#### 2.3.4 Electrical Conductivity

A BK391A digital multimeter was used to measure the resistance over the whole length of the samples. The whole-length resistance was measured at a length of 11 cm of the samples by putting the two probes of the multimeter on the two ends of the pattern. The ends of the samples were covered by aluminum tape, and the sides were properly glued with silver paste to ensure proper connection of the tape to the surface of the coated patterns. This was done to ensure that the measured resistance was representative of the whole length of the sample. In addition, the spot-by-spot electrical conductivity and resistance of the samples were assessed by a four-point probe system (Ossila, UK). In this method, the four points of the probe were placed on different parts of the sample. For each sample, the measurement was done on 10 spots of the sample, and the mean values were reported. The samples were investigated via two separate methods to show the correlation between the results. In addition, the presence of two different measuring methods gave a better perspective for the comparison of the samples.

#### 2.3.5 Contact Angle Measurements

The surface wettability of the coated textile samples was assessed by measuring the water contact angle (WCA). The textile samples were cut into uniform sections to fit the measurement stage and ensure consistent testing conditions. WCA measurements were performed using a Theta Flex optical tensiometer from Biolin Scientific. A 5.0 μL water droplet was gently deposited onto the sample surface at a controlled drop rate of 2.0 μL/s using a microsyringe. The droplet was positioned centrally on the sample to avoid edge effects and ensure uniform measurement conditions. The contact angle was immediately measured upon droplet placement. Images of the droplet were captured at 1 s intervals for a duration of 60 s using the built-in camera. Five droplets were placed on different locations of each sample, and the average value with a standard deviation was reported accordingly.

#### 2.3.6 Electrical Heating Measurement

The Joule heating effect of the coated samples was evaluated in the climate chamber with a constant relative humidity of 21% and a temperature of 21 ± 1 °C. The two sides of the samples were covered with aluminum foil tapes, and the sides of the tape were glued with silver paste to ensure the proper connection of the aluminum to the surface of the samples. Direct current (DC) power supply HY3005D was connected to the two sides of the sample. The heating was done for 90 s followed by rapid cooling after turning off the power source. The surface temperature change of the samples was recorded and analyzed by the FLIR infrared (IR) camera e60 with a temperature range of −20 to 650 °C, a measurement accuracy of 2%, and a thermal sensitivity of <0.05 °C. The camera was fixed at a constant distance from the sample stage, and the distance was determined to cover the whole length of the sample. Furthermore, an angle of 25° between the camera angle of view and the horizontal plane was set to decrease IR reflection from the camera lens. Each test was repeated on three specimens.

#### 2.3.7 Durability

##### 2.3.7.1 Washing Fastness

The washing fastness properties of the coated samples were investigated using a Testex TF418E instrument (China). This device contains a temperature-controllable water bath and 12 rotatable container stages for stainless-steel cylinders with dimensions of 75 ± 5 mm diameter and 125 ± 10 mm height and a capacity of 550 ± 50 mL. The stage rotates at a speed of 40 ± 2 rpm, and the water bath temperature can be controlled from 0 to 100 °C.

The washing procedure followed the ISO 105-C10:2006 standard. According to this standard, the soap solution contained 5 g of soap per liter of water, the liquor to sample mass ratio was 50:1, the water bath temperature was set to 40 °C, and the washing time was 30 min. The samples underwent five washing cycles, with the resistance and weight change evaluated after each cycle. The weight of each sample was measured before and after each washing cycle by using a standard laboratory balance (±0.001 g accuracy) to evaluate any mass loss associated with the washing process. The resistance change was monitored through the whole length of the sample before and after the washing cycle for each sample using a digital multimeter BK391A.

##### 2.3.7.2 Rubbing Fastness

The rubbing fastness properties of the coated samples were evaluated using a Testext TF411 Electronic Crockmeter (China). The coated samples were subjected to 10 back-and-forth rubbing strokes according to the ISO 105-X12:2016 standard method with a Cotton Lawn rubbing cloth ISO 105 F09, and the resistance change was monitored and reported after completion of the 10 cycles.

##### 2.3.7.3 Repeated Bending Cycles

The bending performance of the coated samples was evaluated by monitoring changes in the electrical resistance after multiple bending cycles. The samples were manually folded between two fingers to a 180° angle and then released, with bending applied manually to mimic normal body movements. Resistance across the entire length of each sample was measured after 100, 200, and 300 bending cycles. Three replicates were tested for each sample.

## 3 Results and Discussion

### 3.1 Single-Binder System

#### 3.1.1 Surface Properties

The surface morphology of the coated samples with two different binder setups was analyzed in Figure 1a,b. It can be seen that for the samples containing the A-5001 binder, separated islands were created on the surface, while for the samples coated with the biobinder, a continuous coating without visible separate islands was achieved. Moreover, quantitative analysis of the SEM images with ImageJ indicated that the A-5001 sample exhibited numerous small, dispersed agglomerates covering a large portion of the surface area. In contrast, the biobinder sample showed only a few large agglomerates with a significantly larger average area, covering most of the surface (Table S2). This reflects the formation of larger, interconnected networks in the biobinder system versus fragmented, island-like structures in the A-5001 system. In addition, the SEM images propose a rougher surface for the coated samples with A-5001, while a smoother surface can be seen in the coated samples with the biobinder. Several factors could have contributed to the superior film-forming ability of the biobinder solution. First, the compatibility between the MWCNT particles and the biobinder matrix likely plays a key role; the biobased binder may facilitate better dispersion and interfacial interaction due to its chemical affinity with carbon-based fillers. Second, the viscosity of the biobinder formulation was slightly lower than that of the PU binder, which may have allowed for more uniform spreading and better wetting of the hydrophilic base fabric. Additionally, the biobinder itself is hydrophilic, which likely enhances its adhesion and film formation on the similarly hydrophilic viscose substrate, in contrast to the more hydrophobic PU binder. Water wettability is one of the important surface properties in smart textiles, and it is assessed through WCA measurements. Figure 1c shows the WCA of the coated samples according to the binder type and the MWCNT concentration. The base cellulose fabrics were highly hydrophilic, quickly absorbing water droplets due to the fabric’s porous structure and the hydroxy groups in the cellulose polymer chains.^46^ The coated samples exhibited different surface properties compared to the uncoated fabric as the deposition of the coating ink formed a continuous layer over the fabric’s porous structure. This modification altered the fabric–air and fabric–liquid interfaces, reducing the surface porosity and increasing the WCA, thereby enhancing hydrophobicity without affecting the intrinsic physicochemical properties of the underlying fibers. The samples coated with the ink containing the A-5001 binder became hydrophobic, exhibiting a WCA of around 120°. In contrast, the samples coated with the ink containing the biobinder demonstrated higher wettability, with a WCA of approximately 80°. While the increase in the MWCNT concentration did not yield a statistically significant change in the WCA, a slight upward trend was observed. This trend may be related to the inherent hydrophobicity of carbon-based particles and the additional microroughness introduced by the coating layer. In addition, Figure 1d compares the impact of increasing the coating layer for both of the binders from two to four layers. It can be seen that the WCA stayed nearly constant for both the binders with an increase in the number of coating layers.

**Figure 1:**
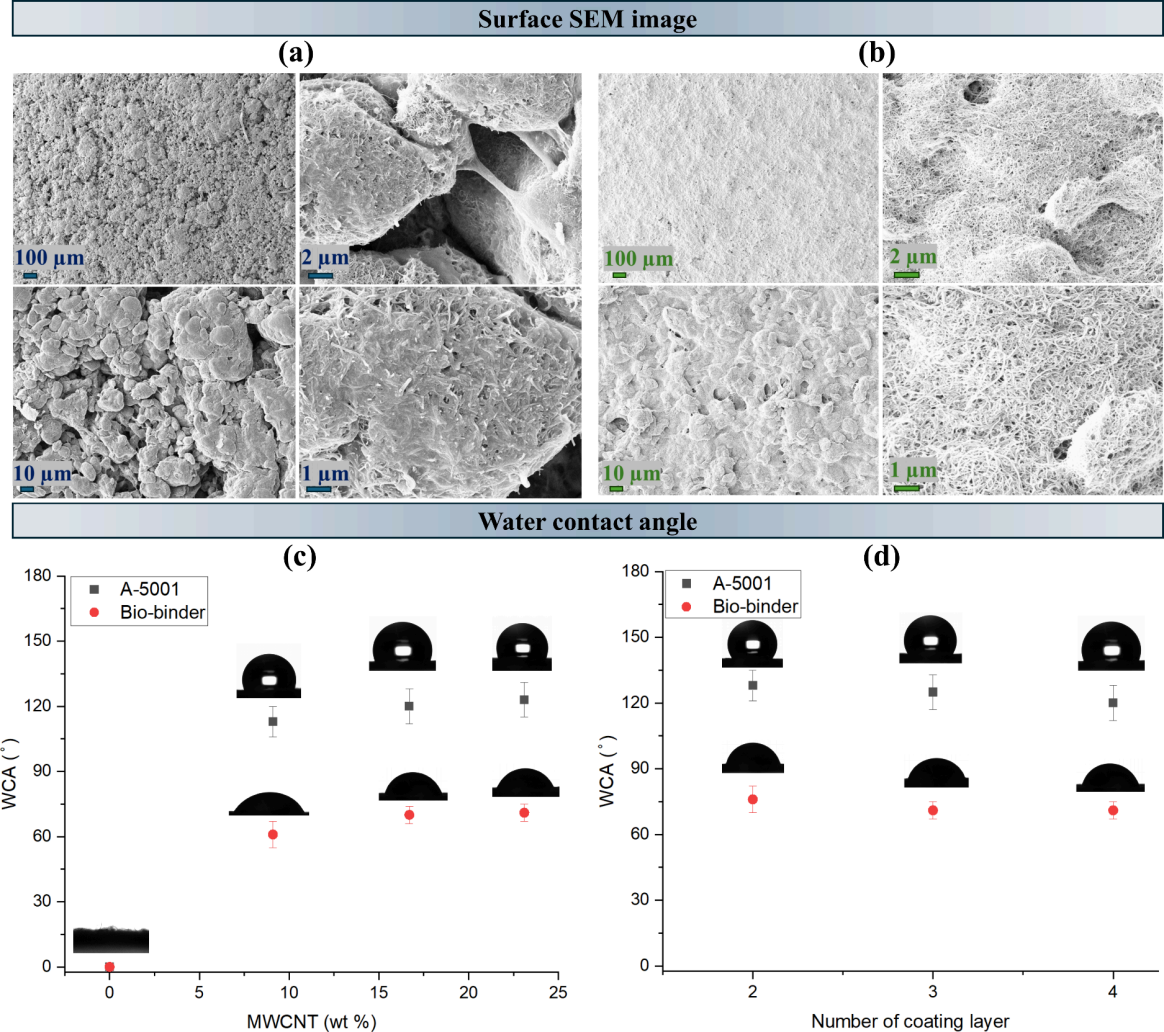
SEM images of the coated fabric containing (a) A-5001 and (b) biobinder. The WCA comparison for two binder systems by varying the (c) MWCNT concentration and (d) number of coating layers.

#### 3.1.2 Electrical Properties

A critical aspect of developing smart textiles for wearable electronics applications is electrical conductivity.^47^ Consequently, the whole-length resistance, mean sheet resistance, and electrical conductivity of the coated samples were measured and compared according to the type of binder, the concentration of MWCNTs, the width of the rectangular coating layer, and the number of coating layers. Cellulose fabric indicates an intrinsic electrical insulator with an electrical conductivity of 2.78 × 10^–9^ S/m. However, applying conductive ink on the fabric altered it to electrically conductive textiles suitable for smart textile applications. Figure 2a–c compares the whole-length resistance, spot-by-spot mean sheet resistance, and spot-by-spot mean conductivity of the coated samples according to the type of binder, the concentration of MWCNTs, and the length of the fabrics. It can be seen that the whole-length resistance is in complete correlation with the spot-by-spot mean sheet resistance and conductivity. The coated samples with the coating ink containing the biobinder showed lower resistance and higher electrical conductivity than those with the coated ink containing the A-5001 binder. This can be due to the presence of separate islands for the samples containing the A-5001 binder, while for the samples coated with the biobinder, a continuous coating without visible separate islands was achieved (Figure 1a,b). The presence of separated islands on the surface of the coated patterns with A-5001 can reduce the number of available channels for the electrons and phonons to move inside the coating layer, while the coated patterns with the biobinder showed a continuous network and more available channels for passage of the electrons and phonons, reducing the resistance of the whole coating layer.^48^ In addition, an increase in the MWCNT concentration resulted in lower resistance and higher electrical conductivity in both the binders. This could be due to an increase in the number of conductive channels inside the coating layer by increasing the MWCNT concentration. Furthermore, the conductivity of the coating patterns was affected by the width of the coating. The patterns with a higher width resulted in lower resistance and higher conductivity. This could be due to the increase in the number of conductive channels by increasing the width. The investigation of the trends of change in resistance and conductivity by changing the number of the coating layers (Figure 2d–f) shows that by increasing the number of coating layers, the resistance decreased, and conductivity increased due to an increase in the number of available conductive channels by increasing the number of layers. This trend was visible in both binder systems. In addition, the trends of the changes in resistance and conductivity with a change in width were repeated for these samples.

**Figure 2:**
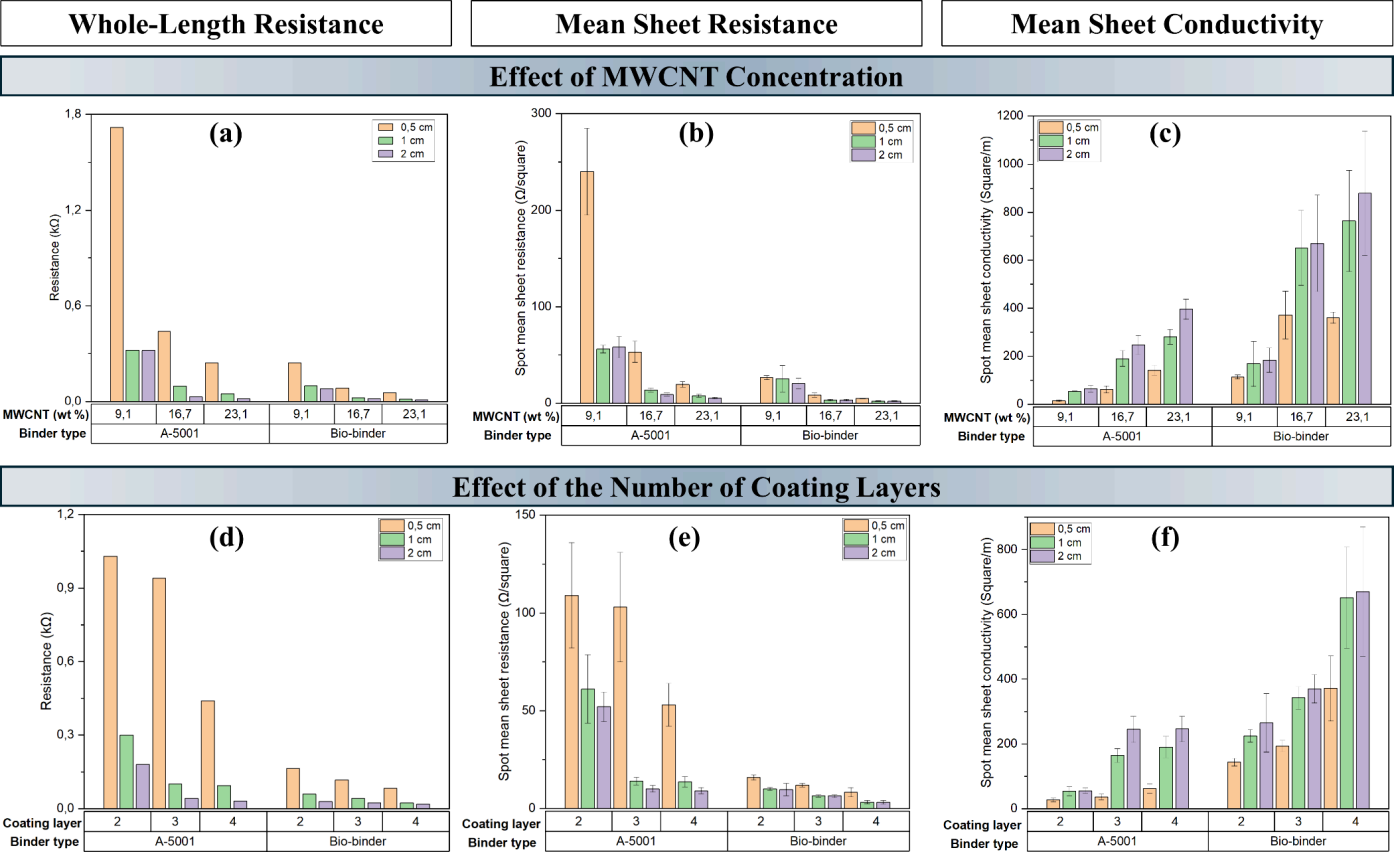
Comparison of (a,d) whole-length resistance, (b,e) spot mean sheet resistance, and (c,f) spot mean sheet conductivity for the two binder systems with varying MWCNT concentrations and number of coating layers.

Investigating the resistance and conductivity data for all sets of samples showed there was a significant change in resistance and conductivity between the widths of 0.5 and 1 cm for all sets of samples. However, it can be seen that the conductivity and resistance were nearly in the same range for most of the samples with a change in width from 1 to 2 cm, while a higher amount of coating ink was required to coat the patterns with a width of 2 cm compared to 1 cm. In addition, the visual investigation of the samples showed a better adhesion for samples with a width of 1 cm compared to 2 cm. As a result, samples with a width of 1 cm were chosen for further investigation.

#### 3.1.3 Electrical Heating Properties

The electrical heating property is affected by the resistance and conductivity of the media. Here, we investigated the electrical heating property for samples of the rectangular pattern with a width of 1 cm and a length of 11 cm. As it was evaluated, the resistance of the samples varied for samples with the type of binder, the concentration of MWCNTs, and the number of coating layers in a constant width. Figure 3a–f compares the Joule heating behavior of 4-layer coated samples with A-5001 and biobinders at MWCNT concentrations of 9.1, 16.7, and 23.1%, while Figure 3g–l examines the effect of coating layers (2, 3, and 4) on Joule heating for samples with the same MWCNT concentration of 16.7%. Both figures illustrate the dynamic electrical heating of the samples at various voltages: 4, 6, 8, 10, and 15 V. As can be seen, the temperature curves contain three stages of initial rapid heating, steady-state, and cooling domain. The electrical heating profiles reveal that the coated samples responded very quickly. The heating temperature trends align with the resistance and conductivity measurements, with samples coated with the A-5001 binder showing lower temperatures compared to those coated with the biobinder. In addition, an increase in the MWCNT concentration, the applied voltage, and the number of coating layers resulted in a higher maximum temperature being achieved. As a result, the O-23.1%-4t sample reached an elevated temperature of 128 °C within 25 s of applying a 15 V voltage, while the A-23.1%-4t sample reached the maximum temperature of 86 °C after 90 s under an applied voltage of 15 V (Figure 3m). However, increasing the number of coating layers beyond three does not significantly affect the electrical heating properties for patterns with the A-5001 binder as similar heating characteristics are observed between A-16.7%-3t and A-16.7%-4t (Figure 3n). In addition, the results indicate that the final temperature of the electrical heating textile increases in a direct relation with applied voltage, consistent with Joule’s law.

**Figure 3:**
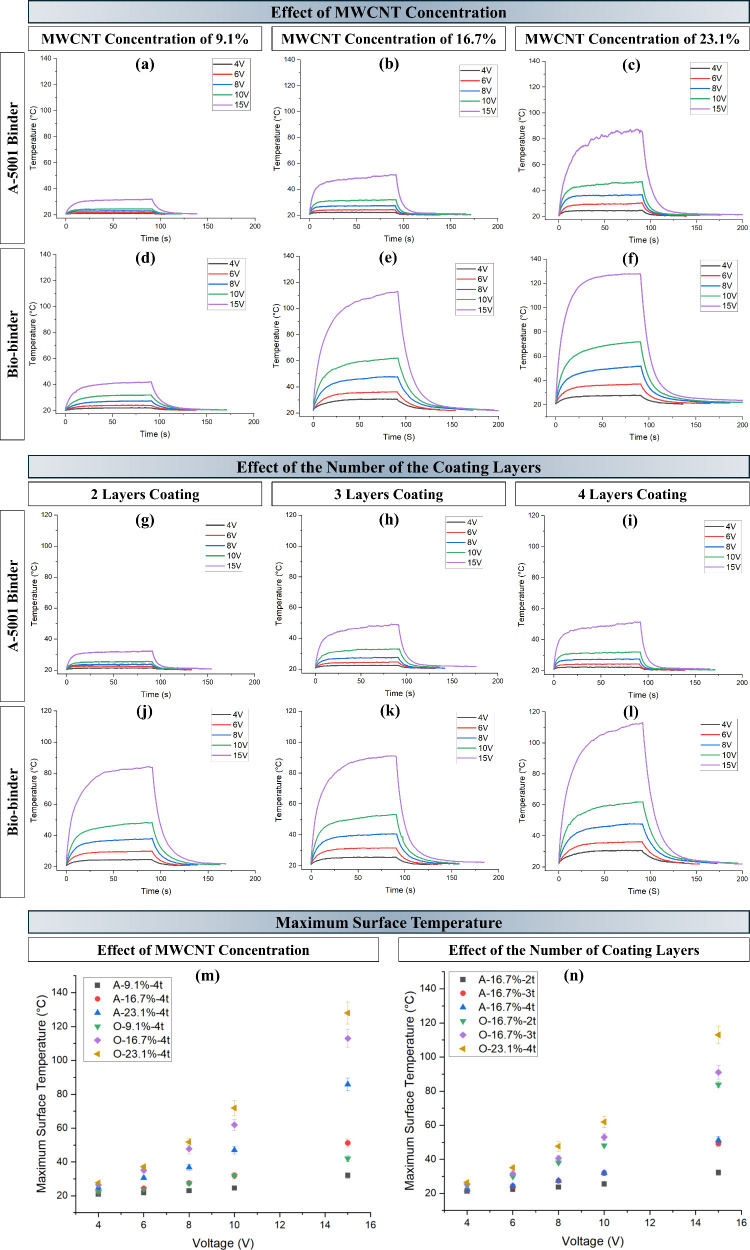
Joule heating behavior comparison for four-layer coated samples with A-5001 binder and the biobinder in a MWCNT concentration of (a,d) 9.1%, (b,e) 16.7%, (c,f) 23.1%; coated samples with the A-5001 binder and the biobinder in a MWCNT concentration of 16.7% and (g,j) two times, (h,k) three times, (i,l) four times coating layers. Maximum surface temperature for (m) four-layer coated samples with the A-5001 binder and the biobinder in different MWCNT concentrations and (n) coated samples with the A-5001 binder and the biobinder in a MWCNT concentration of 16.7% with different numbers of coating layers.

#### 3.1.4 Washing Fastness

Figure 4 shows the effect of the binder type, the MWCNT concentration, and the number of coating layers on the washing speed of the coated patterns. The effect of these factors on washing fastness properties was assessed by observing the change in whole-length resistance and monitoring the weight change after every washing cycle. Resistance changes revealed that patterns coated with inks containing the A-5001 binder were more durable against repeated washing cycles, especially with higher MWCNT concentrations and additional coating layers, compared to those using the bio binder. It can be seen that in the patterns coated with biobinder-containing inks, the coated pattern started to break in several parts for O-16.7%-4t and O-23.1%-4t after the third wash, while it was showing nearly constant resistance (*p*-values: 0.292, 6.63 × 10^–3^, 0.021) for other samples coated with this binder. In addition, the comparison of the weight change for both systems based on the type of binder showed that the weight of the samples containing the A-5001 binder stayed constant (*p*-values: 0.393, 0.467, 0.085, 0.096, and 0.227) in all five washing cycles while an average weight loss of 20% was seen in the coated sample with the biobinder. The common threshold for a *p*-value is 0.05; when the *p*-value is less than this amount, the difference in data is considered statistically significant. Overall, the A-5001PU binder exhibited significantly better binding properties than the biobinder.

**Figure 4:**
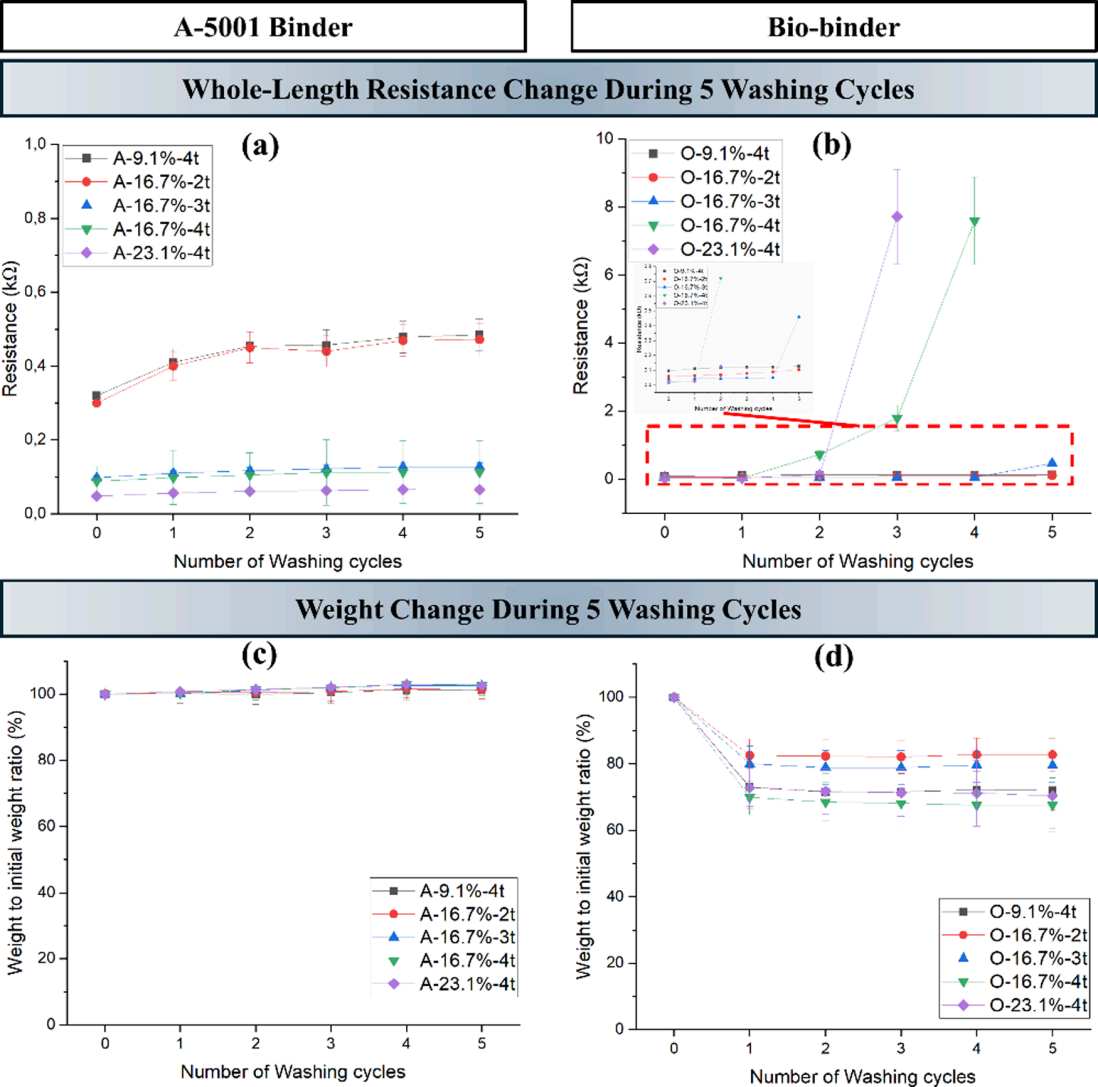
Change in whole-length resistance (a,b) and weight (c,d) for coated samples with A-5001 and biobinder.

#### 3.1.5 Rubbing Fastness

In addition to the washing fastness of smart textiles, rubbing fastness also plays an important role in the challenges of durability of functional particles on textile materials under rubbing conditions. In this respect, the durability of the coated patterns was investigated under 10 cycles of rubbing, and the change in resistance was monitored before and after the rubbing process. Figure 5 shows the change in resistance along the length of samples after 10 rubbing cycles comparing the A-5001 binder with the biobinder. All patterns coated with the biobinder showed an increase in resistance between 30 and 80%. The lowest increase was seen in the O-16.7%-3t sample. However, for the systems containing the A-5001 binder, there was a decrease in resistance for the A-16.7%-3t, A-16.7%-4t, and A-23.1–4t samples. Based on the washing fastness and rubbing fastness properties, A-16.7%-3t and O-16.7%-3t were chosen for further investigation and comparison.

**Figure 5:**
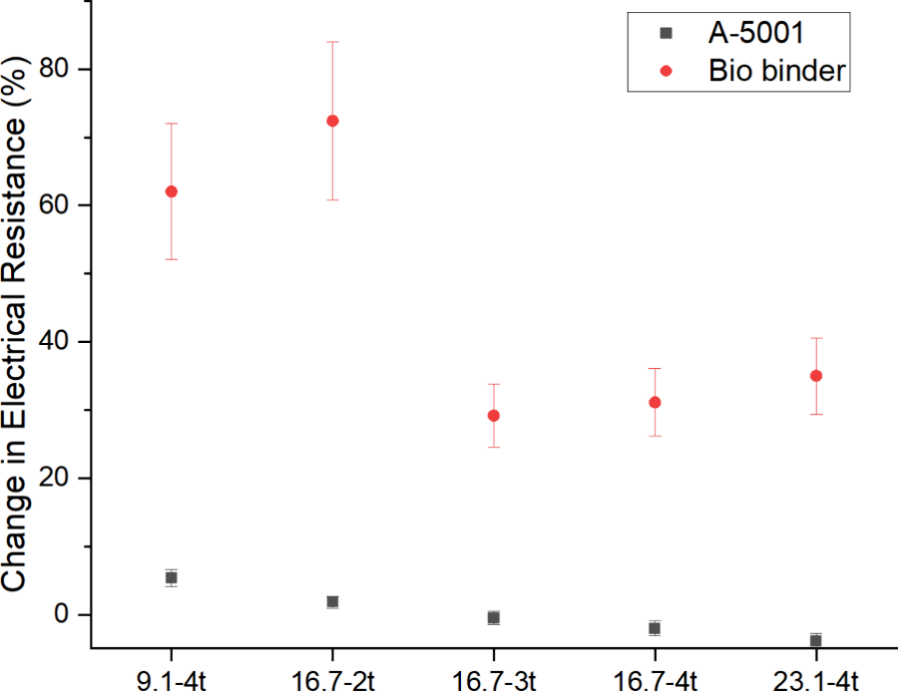
Comparison of whole-length resistance changes in samples coated with A-5001 binder versus biobinder after 10 rubbing cycles.

#### 3.1.6 Morphological Analysis

Figure 6 shows the SEM images of A-16.7%-3t and O-16.7%-3t after 5 washing and 10 rubbing cycles. (a) and (b) show the surface of the sample after five washing cycles. It can be seen that the surface structure and morphology of the coated samples with A-5001 stayed the same, while several breakages on the coating layer of the biobinder-coated samples could be seen after the washing cycles. The morphological integrity of the coating plays a critical role in maintaining a stable conductivity. In the A-5001 coated samples, the continuous hydrophobic binder matrix (Figure 1c,d) prevented water absorption during laundering, thereby avoiding swelling and crack formation. As a result, the conductive MWCNT network remained intact and stable. In contrast, the biobinder coating was inherently hydrophilic (Figure 1c,d), which promotes water uptake during washing. The absorbed water caused swelling and created shrinkage upon drying, resulting in cracks and breakages within the coating. The effect of these can be seen in Figure 4 where the conductivity of the coated pattern with A-5001 stayed nearly constant, while the conductivity of O-16.7%-3t increased more than 10 times after the fifth cycle of washing. (c) and (d) show the surface of the coated samples after 10 rubbing cycles. The morphological analysis of the surface of the A-16.7%-3t sample after rubbing cycles showed a flattened surface where the separated islands were compressed and connected. In other words, voids in the coating may allow conductive MWCNTs to migrate and fill these gaps during repeated rubbing cycles. This phenomenon can increase the number of available channels for the passage of electrons and phonons on the surface of the coated layer. This could be the reason for the decrease in resistance for the coated patterns with the A-5001 binder after 10 cycles of rubbing (Figure 5). A similar morphological event can be seen for O-16.7%-3t, which could result in an increase in the number of available channels for the passage of electrons and phonons. However, several breakages can be seen on the surface of this sample after rubbing cycles, which could reduce the number of conductive channels. This counteracting mechanism resulted in a smaller increase in resistance for O-16.7%-3t after 10 cycles of rubbing compared with after 5 cycles of washing for the same sample.

**Figure 6:**
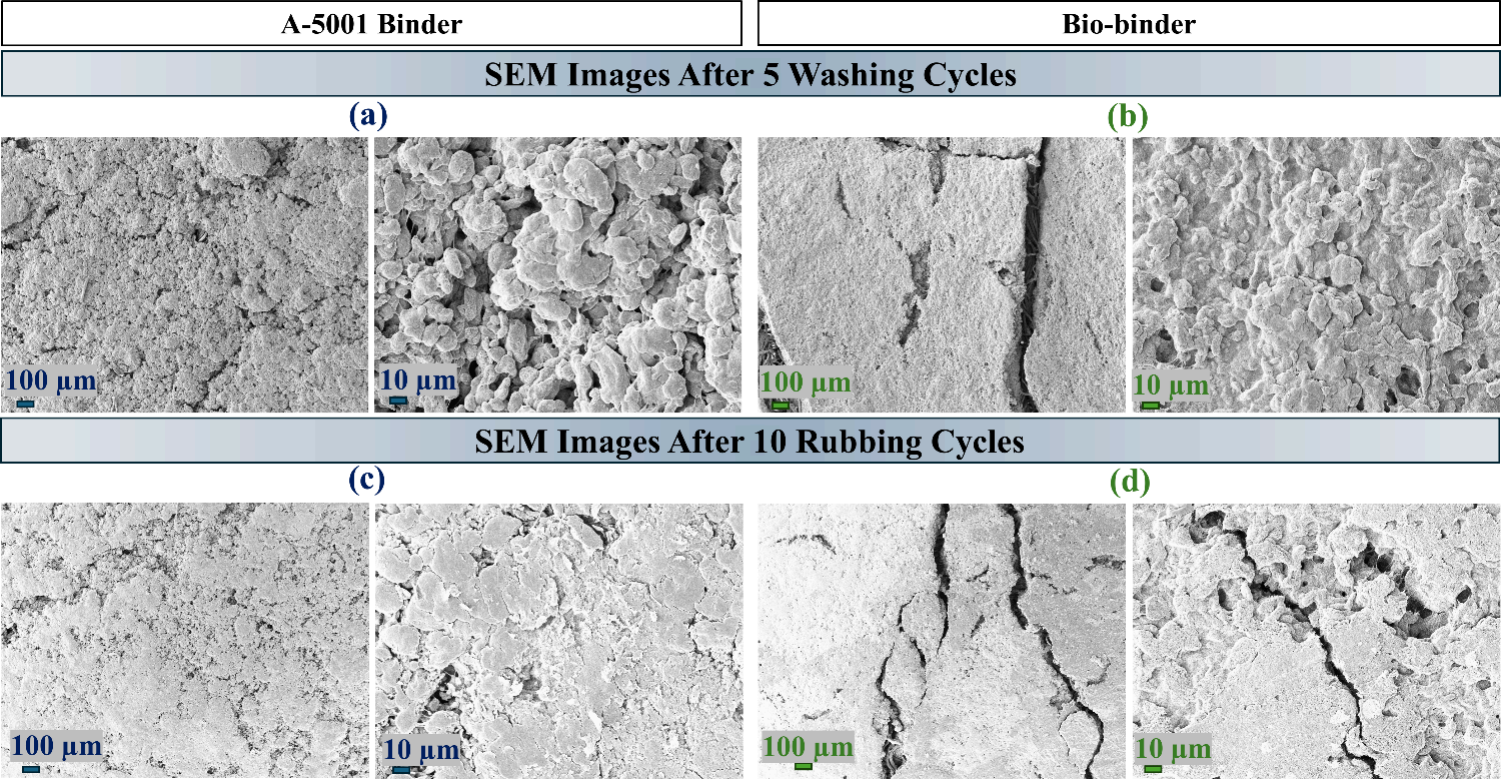
SEM images of (a) A-16.7%-3t and (b) O-16.7%-3t after 5 washing cycles and (c) A-16.7%-3t and (d) O-16.7%-3t after 10 rubbing cycles.

#### 3.1.7 Electrical Heating Properties after Washing and Rubbing Cycles

In order to investigate the impact of washing and rubbing cycles on the efficiency and performance of the coated samples, Joule heating was analyzed for samples A-16.7%-3t and O-16.7%-3t. Figure 7 shows a comparison of the electrical heating properties for two binder systems after washing and rubbing cycles. (a) and (b) compare the temperature–time curves in the two binder systems. The comparison of these graphs with Figure 3h,k revealed a nearly similar heating pattern with a small decrease in the maximum temperature achieved in high voltages of 10 and 15 V for coated samples with the A-5001 binder, while the coated sample with the biobinder showed around 20 and 40 °C decrease in maximum temperature achieved under the applied voltages of 10 and 15 V after five cycles of washing. This is directly affected by the increase in resistance after washing cycles (Figure 4a,b). (c) and (d) illustrate the time-dependent temperature curves for two binder systems after 10 rubbing cycles. A completely similar pattern to the initial behavior can be seen for A-16.7%-3t after rubbing cycles. However, O-16.7%-3t showed around 25 and 15 °C decrease in maximum temperature achieved under the applied voltages of 15 and 10 V. These behaviors are also affected by the change in resistance after 10 cycles of rubbing (Figure 5).

**Figure 7:**
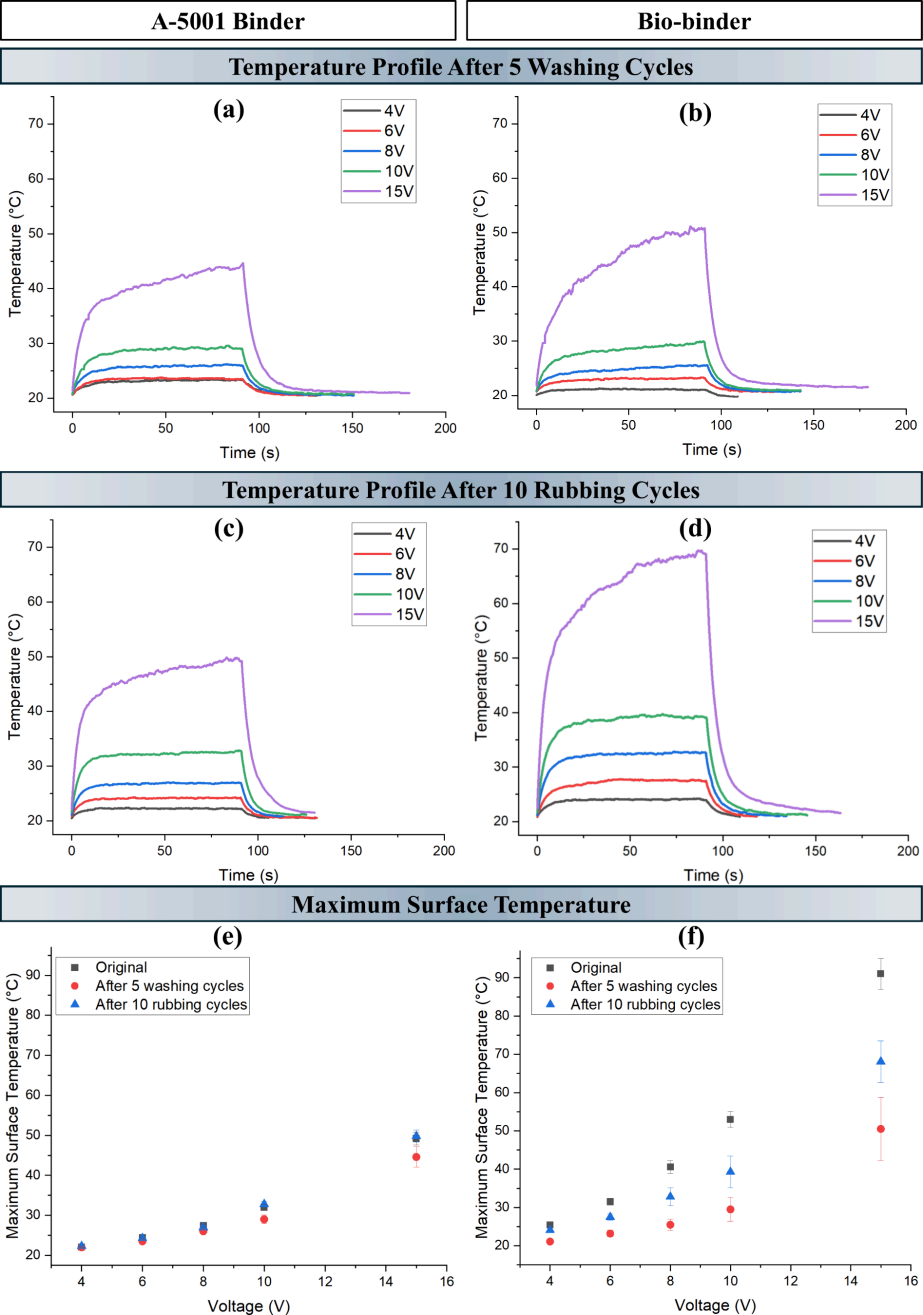
Joule heating curves of (a) A-16.7%-3t and (b) O-16.7%-3t after 5 washing cycles and (c) A-16.7%-3t and (d) O–16.7%-3t after 10 rubbing cycles. Maximum surface temperature change after 5 washing cycles and 10 rubbing cycles in comparison with the original sample for (e) A-5001 based and (f) biobinder-coated samples.

#### 3.1.8 Mechanical Properties

Flexibility is an important aspect of the fabrication of conductive textiles. To assess the flexibility of the coated patterns, bending resistance, bending stiffness, and change in resistance after 100, 200, and 300 cycles of bending were evaluated for A-16.7%-3t and O-16.7%-3t. Figure 8a shows the bending resistance for the two binder systems in bending angles of 0–5°, 0–7.5°, 0–15°, and 0–30°. It can be seen that the samples coated using A-5001 show better flexibility than the coated samples with the biobinder. In addition, the bending stiffness in the measuring angle of 0–7.5° resulted in the bending stiffness of 0.443 ± 0.1 and 0.178 ± 0.027 m.Nm for the samples coated with the biobinder and A-5001, respectively. This indicated the higher bending stiffness and resistance for the samples coated with the biobinder in comparison with the A-5001 binder. Figure 8b exhibits a change in resistance after 100, 200, and 300 bending cycles for two different binder systems. It can be seen that the resistance stayed constant even after 300 bending cycles for the coated samples in the presence of A-5001 binder while the resistance of the coated sample with the biobinder increased around 100% after 300 bending cycles. This could be due to higher bending and resistance stiffness for the coated samples with the biobinder which can result in the production of cracks on the coating layer after multiple bending cycles. The production of cracks on the surface of the coating layer can induce the number of available channels for the passing of electrons and phonons and can result in an increase in the whole-length resistance.

**Figure 8:**
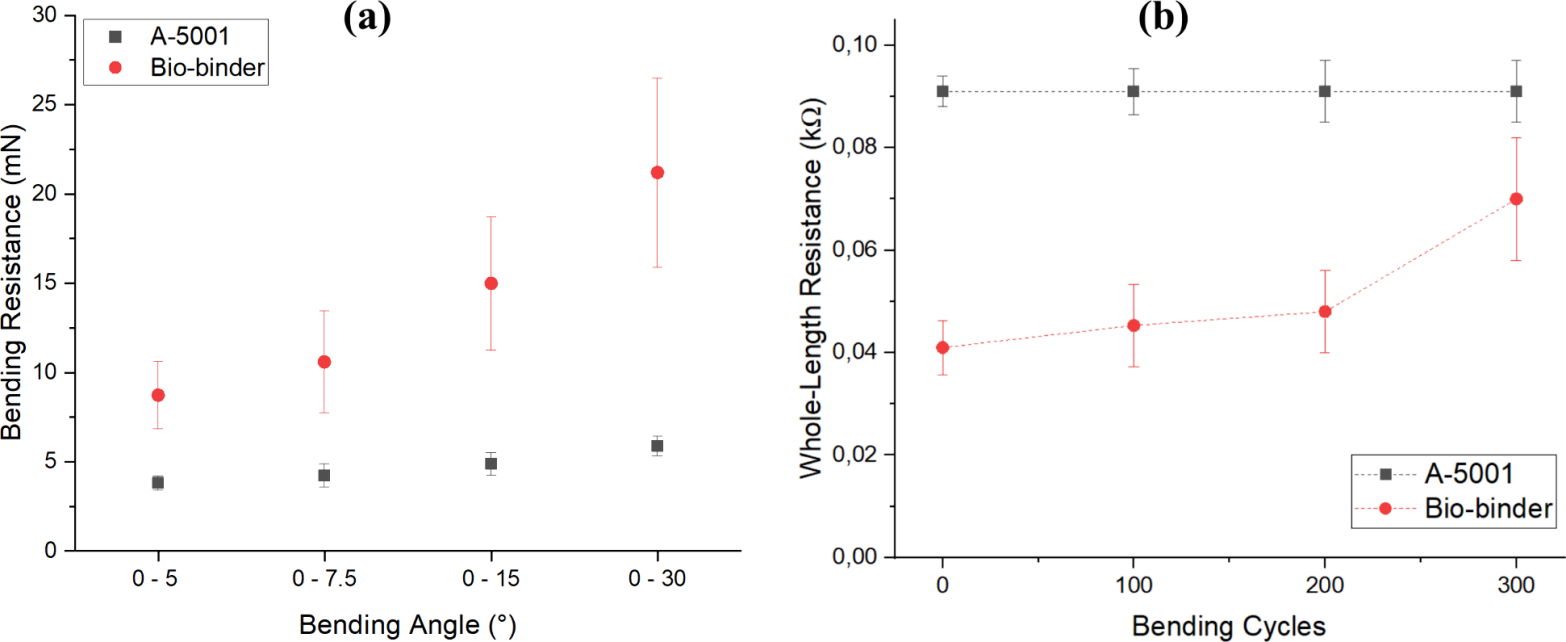
(a) Bending resistance comparison in different bending lengths. (b) Whole-length resistance change after 100, 200, and 300 bending cycles for A-16.7%-3t and O-16.7%-3t samples.

It can be concluded that the A-5001 binder provides better flexibility and resistance stability compared to the biobinder. This makes A-5001 a more reliable option for maintaining consistent conductivity and durability in flexible, conductive textiles.

A thorough analysis of the whole-length resistance, the mean sheet conductivity, Joule heating properties, washing and rubbing fastness, bending resistance and stiffness, and finally resistance durability under bending cycles indicated a lower resistance, higher conductivity, and therefore better joule heating behavior for the coated samples with the biobinder, while they lacked fastness properties and flexibility. Therefore, the hybrid effect of the two coating inks was investigated to address the shortcomings of each with a new approach.

### 3.2 Two-Coating Ink Hybrid Pattern

New sets of samples were prepared using A-16.7% for the first and third coating layer to guarantee the fastness properties, durability, and flexibility and using O-16.7% for the middle layer to improve the conductivity and electrical heating properties. The new samples were named hybrid. Figure 9 shows the properties of the produced sample and compares it to A-16.7%-3t and O-16.7%-3t. The produced sample displayed a WCA of 91° (Figure 9a) compared to 125° for A-16.7%-3t and 71° for O-16.7%-3t, indicating partial mixing of the two binders. This could be understood by comparing the surface of A-16.7%-3t and O-16.7%-3t (Figure 1a,b) with the hybrid sample surface, probably due to the hot-pressing process. The *p*-values indicated statistically significant differences between A-5001 and hybrid (*p* = 0.0061), A-5001 and biobinder (*p* = 0.0006), and hybrid and biobinder (*p* = 0.0331). These results confirm that each binder configuration leads to distinct surface wettability. Figure 9a also shows the electrical properties of the new printed pattern. The differences in whole-length resistance between A-5001, hybrid, and biobinder samples were statistically significant (*p* < 0.005). Specifically, the hybrid sample exhibited a significantly lower resistance than A-16.7%-3t (*p* = 0.0007) while maintaining higher resistance compared to the O-16.7%-3t sample (*p* = 0.0047), reflecting its intermediate conductivity performance. So, we obtained conductive samples with better flexibility and durability by this approach.

**Figure 9:**
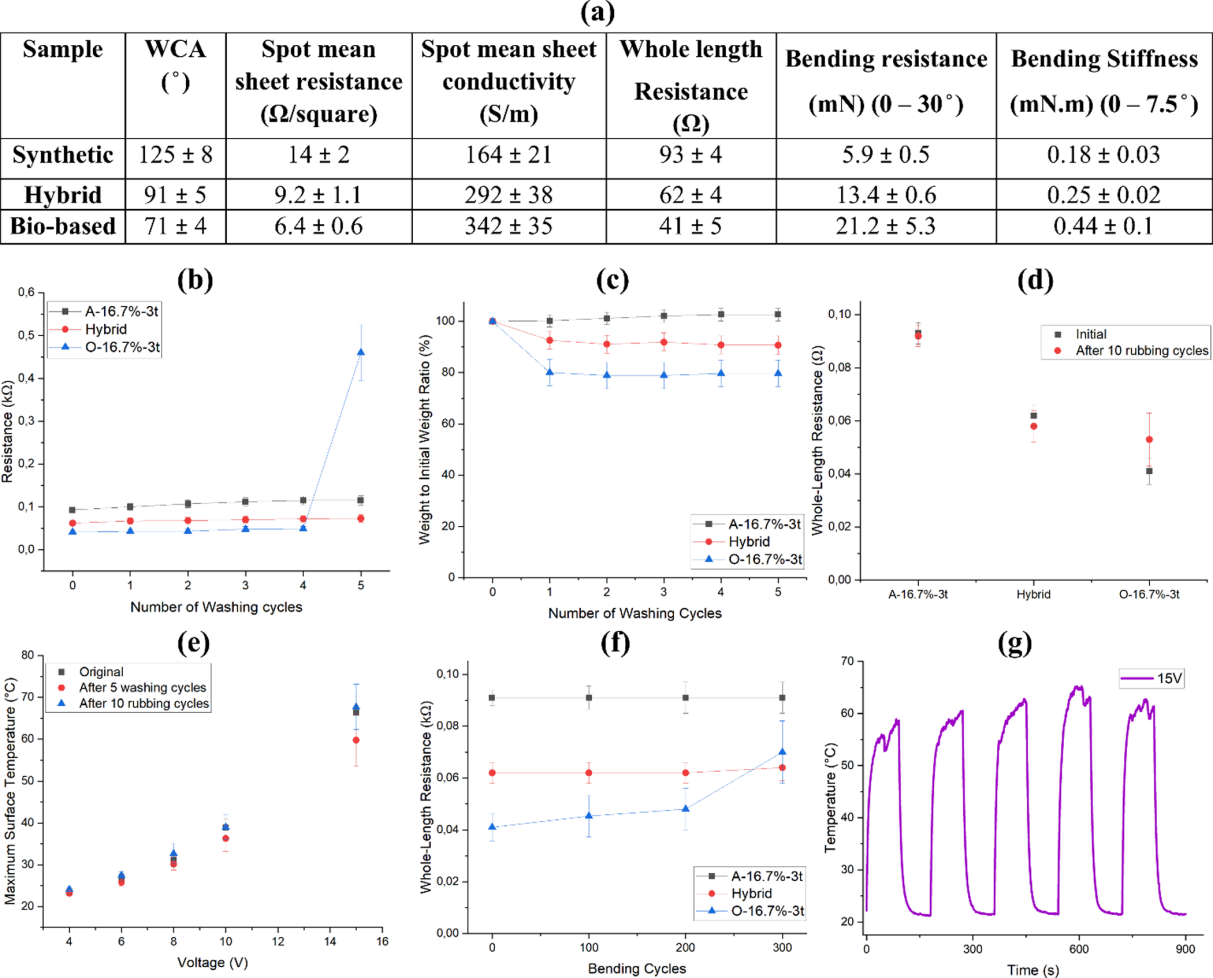
(a) Comparison table of the hybrid sample with A-16.7%-3t and O-16.7%-3t. (b) Whole-length resistance change and (c) weight change of the hybrid sample during five washing cycles compared to A-16.7%-3t and O-16.7%-3t. (d) Whole-length resistance change of the hybrid sample after 10 rubbing cycles. (e) Maximum surface temperature of the hybrid sample after 5 washing cycles and 10 rubbing cycles with the original sample. (f) Whole-length resistance change of the hybrid sample after 100, 200, and 300 bending cycles. (g) Five times washed hybrid sample temperature–time cyclic curve.

The fastness results showed a 6.5% decrease in the whole-length resistance after 10 cycles of rubbing and a 18% increase in the whole-length resistance after 5 cycles of washing for the hybrid sample which showed a great improvement in fastness properties compared to O-16.7%-3t with 30 and 1021% increase in resistance after 10 cycles of rubbing and 5 cycles of washing (Figure 9b). In addition, Figure 9c compares the weight change of the hybrid sample to A-16.7%-3t and O-16.7%-3t during the five washing cycles. The hybrid sample showed a milder reduction in the weight during the washing cycles compared to O-16.7%-3t. Figure 9d compares the rubbing durability of the hybrid sample with A-16.7%-3t and O-16.7%-3t by analyzing the whole-length resistance before and after 10 rubbing cycles. The presented graph showed higher durability for the hybrid sample compared to O-16.7%-3t. In addition, a small decrease in the whole-length resistance can be seen for the hybrid sample after 10 rubbing cycles compared to the initial value which could be due to the softening of the surface and increasing the number of conducting channels. The maximum surface temperature under different applied voltages (Figure 9e) demonstrated an increase in the maximum temperature achieved under the same applied voltages for the hybrid sample compared to A-16.7%-3t. The hybrid sample reached the maximum temperature of 66 °C compared to 49 and 91 °C for A-16.7%-3t and O-16.7%-3t under the applied voltage of 15 V (Figure 7e,f). The maximum surface temperature profile for the hybrid sample showed a small deterioration in Joule heating behavior after 5 washing cycles where a drop of 2.5 and 6.5 °C in maximum temperature achieved under the applied voltages of 10 and 15 V was monitored compared to the unwashed sample. Meanwhile, it can be concluded that after 10 rubbing cycles, the Joule heating behavior stayed nearly constant with a small increase in maximum temperature achieved under high applied voltages of 10 and 15 V. This highlights the hybrid approach’s ability to maintain the Joule heating performance of the coated pattern under fastness conditions. The surface morphology of the hybrid sample after printing, after 5 washing cycles, and after 10 rubbing cycles is shown in Figure S3. It can be seen that both the binders are present on the surface of the printed sample. This could be understood by comparing the surface of A-16.7%-3t and O-16.7%-3t (Figure 1a,b) with the hybrid sample surface, probably due to the hot-pressing process. The investigation of the surface of the hybrid sample after 5 washing cycles and 10 rubbing cycles showed no signs of breakage on the surface and revealed a similar surface morphology as the initial sample before washing and rubbing cycles.

Figure 9f illustrates the trend of change in whole-length resistance during the bending cycles, where the hybrid sample showed a constant resistance even after 300 bending cycles. This trend showed a higher durability for the hybrid sample in comparison with O-16.7%-3t which showed inferior bending durability. This can be affected by the bending resistance and stiffness of the patterns. In this respect, the bending resistance and stiffness results revealed a lower resistance and stiffness in bending which means higher flexibility for the hybrid sample compared to O-16.7%-3t. This higher flexibility helped the hybrid sample preserve the conducting channels even after 300 bending cycles. Moreover, a cyclic heating and cooling test was done on the washed sample to investigate the stability of the heating pattern and maximum achieved temperature after five washing cycles (Figure 9g). It can be seen that the washed sample showed a stable temperature regime under the applied voltage of 15 V during the five cycles of the connection and disconnection of the current, and the temperature reached a maximum after applying a voltage and cooled when the current was removed.

These analyses confirmed the successful synergy effect of two conductive inks using the higher conductivity produced by the O-16.7% ink and the better fastness, durability, and flexibility created by the A-16.7% ink.

## 4 Conclusions

In this study, we produced various aqueous-based conductive inks by modifying the binder system and the MWCNT concentration. Consequently, the inks were printed on the fabric in a rectangular shape with three different widths by using a simple knife-edge coating method. A comprehensive evaluation was done of the structure, functionality, and durability of the coated samples with various formulations and the number of printing layers followed by a thorough comparison. Our findings revealed that the patterns printed with the ink containing the biobinder exhibited hydrophilic morphology, lower resistance, higher conductivity, and superior electrical heating properties. However, they demonstrated low washing durability, especially in high concentrations of MWCNT, low rubbing fastness, low durability in multiple bending cycles, and a higher resistance to bending. Conversely, patterns with ink containing the A-5001 binder showed higher resistance, lower conductivity, and lower achieved temperature under applied voltages but exhibited good washing and rubbing fastness, impressive bending durability after multiple cycles, and lower resistance to bending. FE-SEM analysis revealed that patterns coated with ink containing the A-5001 binder remained intact after washing and rubbing, demonstrating superior fastness. In contrast, patterns coated with the biobinder ink exhibited multiple breakages, indicating lower durability.

By integrating the complementary properties of A-5001 and biobinder inks, the hybrid design achieved a balance between durability and conductivity. The outer A-5001 layer ensured superior washing fastness, rubbing resistance, and flexibility, while the inner biobinder layer enhanced electrical conductivity and Joule heating. This dual-layer strategy delivered significant improvements over single-binder systems, demonstrating a viable pathway toward the advancement of aqueous conductive inks and durable e-textiles for wearable electronics.

## Supplementary Material



## Notes

Surface images of the sample, quantitative morphological data from ImageJ using SEM images of the surface of the samples, as well as cross-sectional and surface FE-SEM micrographs in higher magnifications that further illustrate the morphology and coating structure of the samples (PDF)

## References

[1] Koritsoglou O., Theodorakos I., Zacharatos F., Makrygianni M., Kariyapperuma D., Price R., Cobb B., Melamed S., Kabla A., de la Vega F. (2019). Copper micro-electrode
fabrication using laser printing and laser sintering processes for
on-chip antennas on flexible integrated circuits. Optical Materials Express.

[2] Hon K. K. B., Li L., Hutchings I. M. (2008). Direct
writing technology-Advances
and developments. Cirp Annals-Manufacturing
Technology.

[3] Ju B., Kim I., Li B. M., Knowles C. G., Mills A., Grace L., Jur J. S. (2021). Inkjet Printed Textile Force Sensitive
Resistors for
Wearable and Healthcare Devices. Adv. Healthc
Mater..

[4] Moreira I. P., Sanivada U. K., Bessa J., Cunha F., Fangueiro R. (2021). A Review of
Multiple Scale Fibrous and Composite Systems for Heating Applications. Molecules.

[5] Islam M. R., Afroj S., Novoselov K. S., Karim N. (2022). Smart Electronic Textile-Based
Wearable Supercapacitors. Adv. Sci. (Weinh).

[6] Castano L. M., Flatau A. B. (2014). Smart fabric sensors and e-textile technologies: a
review. Smart Materials and Structures.

[7] Duan Y., You G., Sun K., Zhu Z., Liao X., Lv L., Tang H., Xu B., He L. (2021). Advances in wearable
textile-based micro energy storage devices: structuring, application
and perspective. Nanoscale Adv..

[8] Afroj S., Tan S., Abdelkader A. M., Novoselov K. S., Karim N. (2020). Highly conductive, scalable, and
machine washable graphene-based
E-textiles for multifunctional wearable electronic applications. Adv. Funct. Mater..

[9] Wu Y., Mechael S. S., Carmichael T. B. (2021). Wearable e-textiles using a textile-centric
design approach. Acc. Chem. Res..

[10] Ismar E., Kursun Bahadir S., Kalaoglu F., Koncar V. (2020). Futuristic Clothes:
Electronic Textiles and Wearable Technologies. Glob Chall.

[11] Stoppa M., Chiolerio A. (2014). Wearable electronics and smart textiles: a critical
review. Sensors (Basel).

[12] Dulal M., Afroj S., Ahn J., Cho Y., Carr C., Kim I. D., Karim N. (2022). Toward Sustainable
Wearable Electronic
Textiles. ACS Nano.

[13] Wang S., Du X., Luo Y., Lin S., Zhou M., Du Z., Cheng X., Wang H. (2021). Hierarchical
design of waterproof,
highly sensitive, and wearable sensing electronics based on MXene-reinforced
durable cotton fabrics. Chemical Engineering
Journal.

[14] Cherston, J. ; Paradiso, J. A. SpaceSkin: Development of aerospace-grade electronic textile for simultaneous protection and high velocity impact characterization. In Sensors and Smart Structures Technologies for Civil, Mechanical, and Aerospace Systems 2019; SPIE, 2019: Vol. 10970, pp 111–125.

[15] Ojstrsek A., Jug L., Plohl O. (2022). A Review of Electro Conductive Textiles Utilizing the
Dip-Coating Technique: Their Functionality, Durability and Sustainability. Polymers (Basel).

[16] Angelucci A., Cavicchioli M., Cintorrino I. A., Lauricella G., Rossi C., Strati S., Aliverti A. (2021). Smart Textiles and
Sensorized Garments for Physiological Monitoring: A Review of Available
Solutions and Techniques. Sensors (Basel).

[17] Ma R., Li D., Xu C., Yang J., Huang J., Guo Z. (2024). Fabricated
advanced textile for personal thermal management, intelligent health
monitoring and energy harvesting. Adv. Colloid
Interface Sci..

[18] Komolafe A., Zaghari B., Torah R., Weddell A. S., Khanbareh H., Tsikriteas Z. M., Vousden M., Wagih M., Jurado U. T., Shi J. J. (2021). E-Textile Technology
Review-From Materials to Application. Ieee Access.

[19] Shi J., Liu S., Zhang L., Yang B., Shu L., Yang Y., Ren M., Wang Y., Chen J., Chen W. (2020). Smart
Textile-Integrated Microelectronic Systems for Wearable Applications. Adv. Mater..

[20] Ali A., Ashfaq M., Qureshi A., Muzammil U., Shaukat H., Ali S., Altabey W. A., Noori M., Kouritem S. A. (2023). Smart Detecting
and Versatile Wearable Electrical Sensing Mediums for Healthcare. Sensors (Basel).

[21] Islam R., Khair N., Ahmed D. M., Shahariar H. (2019). Fabrication
of low cost and scalable carbon-based conductive ink for E-textile
applications. Materials Today Communications.

[22] Bayram Y., Zhou Y., Shim B. S., Xu S., Zhu J., Kotov N. A., Volakis J. L. (2010). E-textile conductors
and polymer
composites for conformal lightweight antennas. IEEE Transactions on Antennas and Propagation.

[23] Han J.-W., Meyyappan M. (2011). Copper oxide
resistive switching memory for e-textile. AIP
Adv..

[24] Xu L. Y., Yang G. Y., Jing H. Y., Wei J., Han Y. D. (2014). Ag-graphene
hybrid conductive ink for writing electronics. Nanotechnology.

[25] Irimia-Vladu M., Glowacki E. D., Voss G., Bauer S., Sariciftci N. S. (2012). Green and
biodegradable electronics. Mater. Today.

[26] Kamyshny A., Magdassi S. (2014). Conductive nanomaterials for printed
electronics. Small.

[27] Morsada Z., Hossain M. M., Islam M. T., Mobin M. A., Saha S. (2021). Recent progress
in biodegradable and bioresorbable materials: From passive implants
to active electronics. Applied Materials Today.

[28] Remeika M., Qi Y. (2018). Scalable solution coating
of the absorber for perovskite solar cells. Journal of energy chemistry.

[29] Dimitriou E., Michailidis N. (2021). Printable
conductive inks used for the fabrication
of electronics: an overview. Nanotechnology.

[30] Tseng M.-L., Tan R. R., Siriban-Manalang A. B. (2013). Sustainable consumption
and production for Asia: sustainability through green design and practice. J. Clean. Prod..

[31] Karana E. (2012). Characterization
of ’natural’ and ’high-quality’ materials
to improve perception of bio-plastics. Journal
of Cleaner Production.

[32] Karana E., Nijkamp N. (2014). Fiberness, reflectiveness and roughness
in the characterization
of natural and high quality materials. Journal
of Cleaner Production.

[33] Van
der Velden N. M., Kuusk K., Köhler A. R. (2015). Life cycle
assessment and eco-design of smart textiles: The importance of material
selection demonstrated through e-textile product redesign. Materials & Design.

[34] Repon M. R., Mikucioniene D. (2021). Progress in Flexible Electronic Textile
for Heating
Application: A Critical Review. Materials (Basel).

[35] Rotzler S., Kallmayer C., Dils C., von Krshiwoblozki M., Bauer U., Schneider-Ramelow M. (2020). Improving
the washability of smart
textiles: Influence of different washing conditions on textile integrated
conductor tracks. Journal of The Textile Institute.

[36] Atalay O., Kennon W. R., Demirok E. (2015). Weft-knitted strain sensor for monitoring
respiratory rate and its electro-mechanical modeling. IEEE Sensors Journal.

[37] Biermaier C., Bechtold T., Pham T. (2021). Towards the
Functional Ageing of
Electrically Conductive and Sensing Textiles: A Review. Sensors (Basel).

[38] Rotzler S., Krshiwoblozki M. v., Schneider-Ramelow M. (2021). Washability of e-textiles: Current
testing practices and the need for standardization. Text. Res. J..

[39] Wang F., Liu Y., Yu J., Li Z., Ding B. (2024). Recent progress on
general wearable electrical heating textiles enabled by functional
fibers. Nano Energy.

[40] Sarkar, M. K. ; Fan, J. Textiles for heat generation. In Functional and Technical Textiles; Elsevier, 2023; pp 397–418.

[41] Xuan X. (2008). Joule heating
in electrokinetic flow. Electrophoresis.

[42] Orban, R. F. ; Lewis, J. C. Electrically heated gloves. Google Patents, 1988.

[43] Zhang L., Baima M., Andrew T. L. (2017). Transforming Commercial Textiles
and Threads into Sewable and Weavable Electric Heaters. ACS Appl. Mater. Interfaces.

[44] Logothetis I., Gkoutzeli D., Kagkas D., Vassiliadis S., Siores E., Pirogova E. (2019). Thermoelectric
Heat Patch for Clinical
and Self-Management: Melanoma Excision Wound Care. Ann. Biomed Eng..

[45] Gressel J., Hoffmann S. (2024). Generation of biobased,
biodegradable non-woven straw
mats for multiple environmentally friendly uses. bioRxiv.

[46] Abdi B., Tarhini A., Baniasadi H., Tehrani-Bagha A. R. (2024). Developing
Graphene-based Conductive Textiles Using Different Coating Methods. Advanced Materials Technologies.

[47] Ouyang Z. F., Li S. H., Liu J. T., Yu H. Y., Peng L. H., Zheng S., Xu D. W., Tam K. C. (2022). Bottom-up reconstruction
of smart textiles with hierarchical structures to assemble versatile
wearable devices for multiple signals monitoring. Nano Energy.

[48] Abdi B., Baniasadi H., Tarhini A., Tehrani-Bagha A. (2025). Enhancing
Electrical Conductivity in Cellulosic Fabric: A Study of Bio-Based
Coating Formulations. Advanced Materials Technologies.

