# Supplementary material for: Toward Robust Electrical Heating Textiles: Factors Influencing Electrical Heating Performance and Durability
Source: ACS Omega. 2025 Oct 20;10(43):51354–67. doi: 10.1021/acsomega.5c06733 (PMC12593063; doi:10.1021/acsomega.5c06733)
Supplement: Supplementary file 1 [file ao5c06733_si_001.pdf]

Supporting information for

Towards Robust Electrical-Heating Textiles: Factors Influencing Electrical Heating Performance and Durability

Babak Abdi[1], and Ali R. Tehrani-Bagha [1*]
[1]School of Chemical Engineering, Aalto University, Espoo 02150, Finland
[*] Corresponding author: ali.tehrani@aalto.fi

List of figures and tables:

Figure S1. Image of the printed patterns.
Figure S2. SEM images of (a) A-16.7%-3t, (b) A-16.7%-3t after washing, (c) A-16.7%-3t after rubbing, (d) O-16.7%-3t, (e) O-16.7%-3t after washing, (f) O-16.7%-3t after rubbing.
Figure S3. SEM images of the hybrid sample after printing, after 5 washing cycles, and after 10 rubbing cycles.
Table S1. Quantitative Morphological Data from ImageJ.

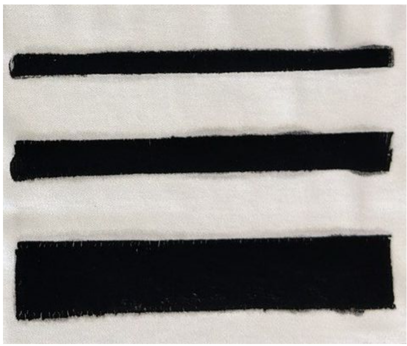

Figure S4. Image of the printed patterns.

Table S2. Quantitative Morphological Data from ImageJ.

Sample	Count	Total area (μm^2)	Average size (μm^2)	% Area Coverage
A-5001	732	7884.6	0.95	81.2
Bio-binder	14	8954.4	639.6	90.2

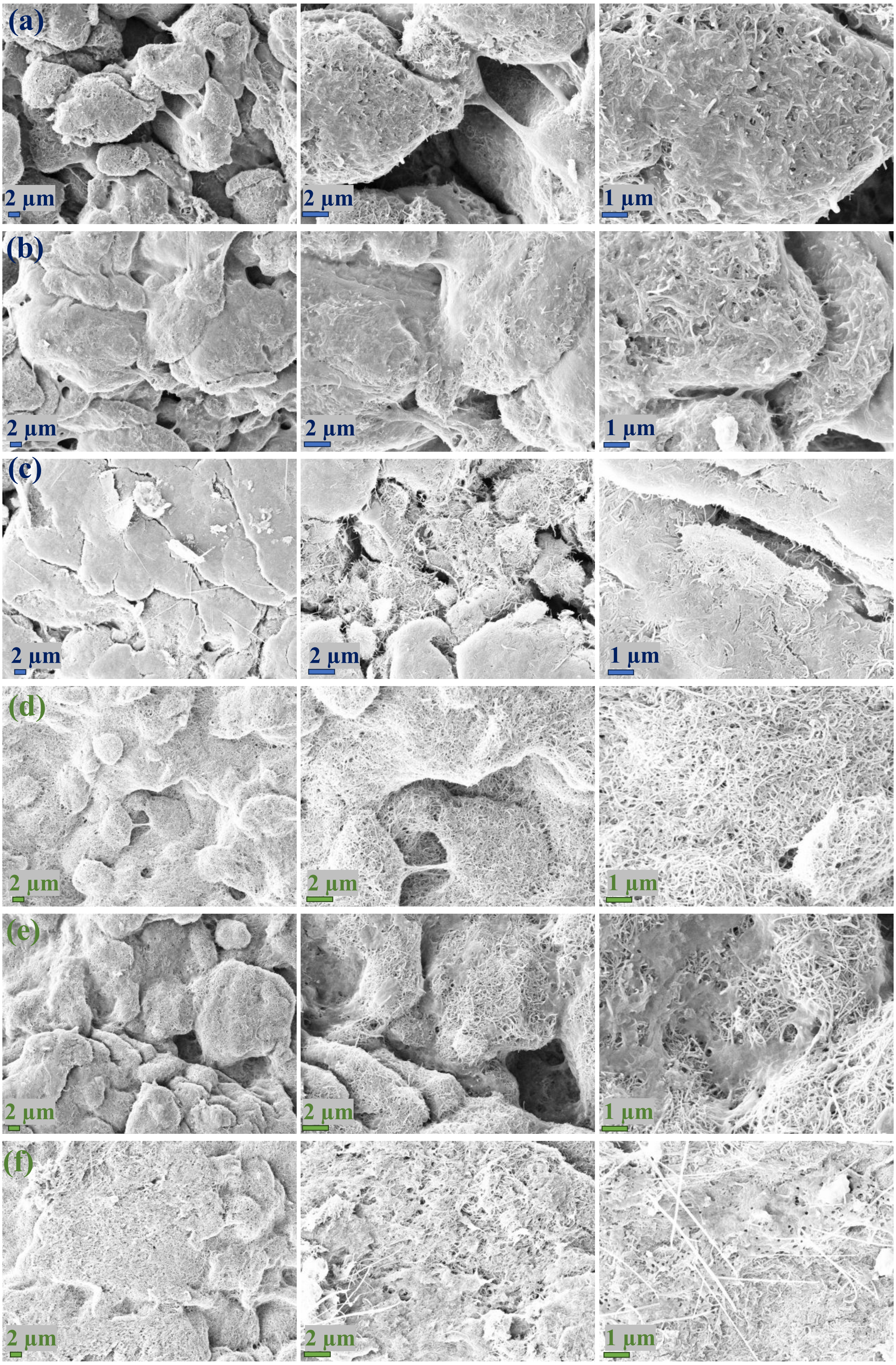

Figure S5. SEM images of (a) A-16.7%-3t, (b) A-16.7%-3t after washing, (c) A-16.7%-3t after rubbing, (d) O-16.7%-3t, (e) O-16.7%-3t after washing, (f) O-16.7%-3t after rubbing.

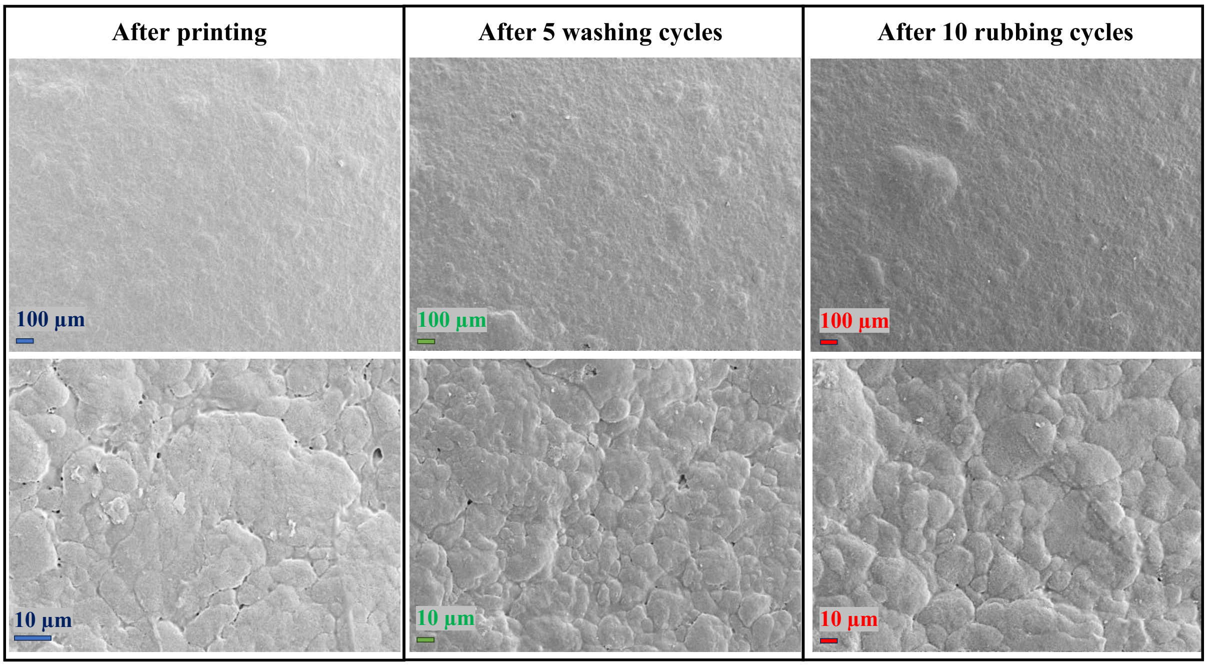

Figure S6. SEM images of the hybrid sample after printing, after 5 washing cycles, and after 10 rubbing cycles.
